# Cyclopædia of the Practice of Medicine

**Published:** 1876-01

**Authors:** 


Cyclopædia of the Practice of Medicine. Edited by Dr. H. Von Ziemssen, Professor of Clinical Medicine in Munich, Bavaria. Volume X., Diseases of the Female Sexual Organs. By Professor Carl Schroeder, of Erlangen, Bavaria. Albert H. Buck, M.D., New York Editor American edition. William Wood & Co, New York. 1875.

The profession will ever feel grateful to William Wood & Co. for the enterprise they have displayed in presenting this great work. No publication for the past half century, in the English language at least, will at all compare with this valuable work. The profession has so long felt the necessity of a condensed, but complete, record of the present status of medical science, particularly in the departments of etiology, pathology, and treatment, that we are not surprised at the great popularity of Ziemssen's Cyclopædia, as it so perfectly fills the want so much felt.

The tenth volume, which has just been received, embraces the diseases of the female sexual organs, and is from the pen of Professor Carl Schroeder, of Erlangen, Bavaria. The limited space that this notice must occupy makes a review at this time impossible. In a future number we hope to be able to give some of the more prominent features of this volume. The text is beautifully illustrated with wood cuts and electrotypes. As will be seen from the following notice, this volume appears out of its proper place, for reasons there assigned:

"Many of the subscribers to this great work are aware that the volumes of the German edition are not being issued in regular succession—some of those treating upon subjects of greatest interest having the precedence, although numbered to conform to the plan of the entire work. It has been concluded to follow the same course with this translation; and in compliance with the expressed wish of many subscribers, volume X., Schroeder's 'Diseases of the Female Sexual Organs,' is now published. Volumes IV. and V. will follow.

"William Wood & Co., Publishers, New York."

